# Highly integrated watch for noninvasive continual glucose monitoring

**DOI:** 10.1038/s41378-022-00355-5

**Published:** 2022-02-23

**Authors:** Tianrui Chang, Hu Li, Nianrong Zhang, Xinran Jiang, Xinge Yu, Qingde Yang, Zhiyuan Jin, Hua Meng, Lingqian Chang

**Affiliations:** 1 Key Laboratory of Biomechanics and Mechanobiology, Ministry of Education, Beijing Advanced Innovation Center for Biomedical Engineering, School of Biological Science and Medical Engineering, Beihang University, Beijing, 100083 China; 2 Department of Biomedical Engineering, City University of Hong Kong, Hong Kong, China; 3 General Surgery Department & Obesity and Metabolic Disease Center, China-Japan Friendship Hospital, Beijing, 100029 China; 4 Sense Future (HangZhou) Co., Ltd, Hangzhou, 311217 China

**Keywords:** Electrical and electronic engineering, Biosensors

## Abstract

This article reports a highly integrated watch for noninvasive continual blood glucose monitoring. The watch employs a Nafion-coated flexible electrochemical sensor patch fixed on the watchband to obtain interstitial fluid (ISF) transdermally at the wrist. This reverse iontophoresis-based extraction method eliminates the pain and inconvenience that traditional fingerstick blood tests pose in diabetic patients’ lives, making continual blood glucose monitoring practical and easy. All electronic modules, including a rechargeable power source and other modules for signal processing and wireless transmission, are integrated onto a watch face-sized printed circuit board (PCB), enabling comfortable wearing of this continual glucose monitor. Real-time blood glucose levels are displayed on the LED screen of the watch and can also be checked with the smartphone user interface. With 23 volunteers, the watch demonstrated 84.34% clinical accuracy in the Clarke error grid analysis (zones A + B). In the near future, commercial products could be developed based on this lab-made prototype to provide the public with noninvasive continual glucose monitoring.

## Introduction

The latest report from the International Diabetes Federation shows that diabetes affected 463 million adults worldwide in 2019, and this number is still increasing. Blood glucose remains the major criterion for the diagnosis and management of diabetes, where fasting blood glucose ≥ 7.0 mmol/L and/or 2 h postprandial blood glucose ≥ 11.1 mmol/L confirm hyperglycemia^1,2^. The traditional fingerstick blood glucose test requires carefully planned performance to catch the peaks and troughs of blood glucose levels, bringing inconvenience and pain to patients’ lives. For diabetic patients, daily health management relies heavily on invasive blood glucose testing multiple times per day, which makes a user-friendly system for painless, automatic, and continuous blood glucose measurement highly desirable^3,4^.

Considerable research efforts have been made in the last decade to develop noninvasive methods for the continuous monitoring of glucose, as well as other biomarkers. (e.g., lactate, uric acid, sodium, and potassium) in biofluids (e.g., sweat, saliva, tear, and ISF), resulting in a variety of devices^5–10^. Microneedles, albeit minimally invasive, are still accompanied by the risk of infection^11–13^, whereas devices such as mouthguards and contact lenses^14–17^ lack well-established evidence for the correlation between saliva or tears and blood glucose. Sweat sensors^18,19^ are the most widely developed strategy for metabolite and electrolyte analysis due to the accessibility of sweat and the safety of the device. However, for the elderly, sick, or disabled whose physical conditions render sweating exercises impractical, sweat sensors lose utility as sweat becomes less available; the amount of sweat on the skin in the resting state is usually insufficient for biomarker detection. Skin ISF, on the other hand, is a stably and abundantly present biofluid regardless of physical conditions. It diffuses from the blood capillaries and supplies nutrients, including glucose, to the surrounding cells, establishing a reliable correlation between blood and ISF glucose levels, and thus is increasingly used as a source of biomarkers. ISF-based glucose monitors developed in recent years have proven reliable and accurate in research studies^20,21^, but their relatively low level of integration limits their application beyond the benchtop. As activity tracking features (e.g., sleep patterns^22,23^, heart rate^24,25^, blood pressure^26,27^, and blood oxygen^28,29^) become increasingly popular in wearable electronics, especially smartwatches and smart bands, ISF-based noninvasive glucose meters could be further consolidated and miniaturized into the form of a watch to better enable continual monitoring. A brief summary and comparison of representative glucose meters based on the same sample extraction mechanism are included in the supplementary material (Table S1).

Herein, we introduce a highly integrated watch for the practical continual monitoring of blood glucose in a painless, noninvasive manner. The watch integrates an LED screen for result display, a printed circuit board (PCB) for signal processing and transmission, a rechargeable battery as a power supply, and a flexible glucose sensor patch fixed on the watchband to detect glucose from the transdermally extracted ISF. To enhance the accuracy of glucose measurement, a calibration algorithm was applied to the data processing of the watch. Additionally, an app was developed to control the watch and receive real-time blood glucose measurement results on a smartphone. Based on these achievements, we believe this watch could provide a great convenience in daily life and contribute to health care in the near future.

## Results and discussion

### Overall system design

**Fig. 1:**
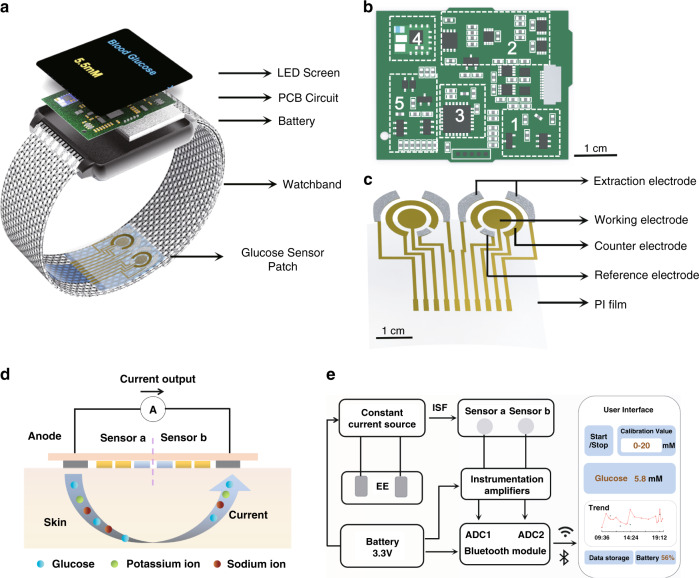
Overall design of the watch for noninvasive continual glucose monitoring. **a** Exploded view of the watch. **b** Diagram of the printed circuit board (PCB) in the watch showing each functional module. (1) Constant current source, (2) A/D differential module, (3) microcontroller, (4) bluetooth module, and (5) power supply. **c** Structure of the flexible glucose sensor patch for interstitial fluid (ISF) extraction and glucose detection. **d** Working mechanism of reverse iontophoresis for noninvasive ISF extraction achieved with the glucose sensor patch. **e** System-level block diagram of the watch and user interface on a smartphone, showing the synergy among the functional units

The overall design of the watch is presented in Fig. 1. The watch consists of an LED screen, a PCB circuit, a rechargeable battery, a circular watchband, and a glucose sensor patch (Fig. 1a). The watch face-sized PCB circuit consolidates five functional modules for system power, signal processing, and wireless transmission, thus delivering a highly integrated wearable electronic system (Fig. 1b). The flexible glucose sensor patch was fabricated on a 100 μm thick polyimide (PI) film with MEMS technology (Fig. S1), which is fixed on the watchband and conformally attaches to the skin. The patch contains two glucose sensors, each consisting of a working electrode, a reference electrode, and a counter electrode (Fig. 1c). Each sensor is also surrounded by a pair of extraction electrodes for the noninvasive extraction of ISF from the skin. Biomarkers are obtained transdermally through reverse iontophoresis (Fig. 1d), which comprises two underlying mechanisms, i.e., electromigration and electroosmosis^30,31^. Upon the application of an electric current through the extraction electrodes, small molecular-sized ions in the ISF under the skin surface electromigrate toward the electrode of opposite polarity. Since skin at physiological pH is negatively charged and thus permselective to positive ions, an electroosmotic solvent flow is induced by the cations (e.g., Na^+^, K^+^) and carries solute species, including neutral and especially polar molecules such as glucose and lactate, to the cathode^32,33^. The extracted glucose is then detected by the nearby sensor.

The system-level overview in Fig. 1e illustrates the synergistic working principle of the glucose monitor. A rechargeable battery (3.3 V) serves as the power supply of the whole system. A constant current source is connected to one end of each extraction electrode to supply the microampere level (50 μA) of electric current required for reverse iontophoresis. The glucose molecules extracted from the ISF are detected by the two sensors, eliciting a current response that is converted to a voltage signal and amplified by the instrumentation amplifier. The voltage signal is then transmitted to the ADC converter, and the consequent digital signal serves as the data input for the microcontroller, which executes the calibration algorithm and calculates the corresponding blood glucose level. Finally, a numerical value is presented on the LED screen of the watch and transmitted to the smartphone user interface via the Bluetooth module. The circuit design is described in detail in the “Methods and materials” section and Figs. S13 and S14.

### Characteristics of glucose sensors

**Fig. 2:**
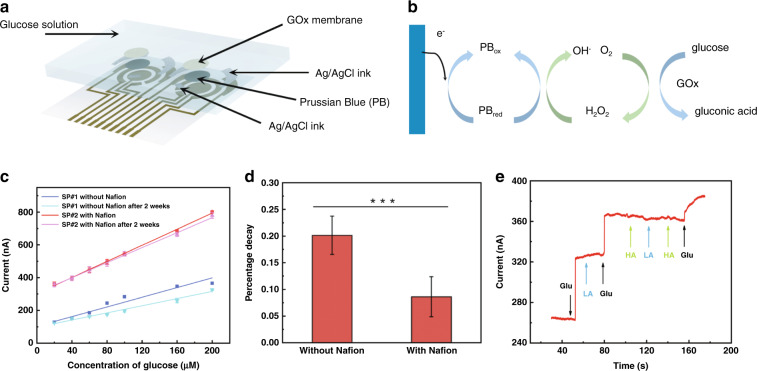
Working principle of the glucose sensor patch and characterization in a semi-infinite diffusion environment. **a** Layer-by-layer diagram of sensor patch components. **b** The two-step mechanism of glucose detection: glucose oxidase (GO*x*)-catalyzed glucose oxidation, yielding H_2_O_2_, and Prussian blue (PB)-catalyzed H_2_O_2_ reduction. The electrocatalyst PB consumes an electron during the reaction, causing an amperometric response. **c** Amperometric responses of glucose sensor patches with (SP#2, 3 replicates) and without (SP#1, 3 replicates) Nafion film in the two-week test, demonstrating the long-term stability of the sensors, especially with Nafion modification. Data represent the mean ± s.d. of three replicates. **d** Comparison of the percentage decrease in sensor sensitivity between SP#1 and SP#2. Data represent the mean ± s.d. of three replicates. ***p < 0.001 by Student’s t-test. **e** Amperometric responses of SP#2 to glucose in contrast to interference components lactic acid (LA) and hyaluronic acid (HA)

The glucose sensor patch was fabricated in the laboratory. After Au was sputter-deposited onto the PI film (see “Methods and materials” for details), the counter electrodes were left as is, while other electrodes were further modified. Ag/AgCl ink was screen printed onto the extraction electrodes and the reference electrodes. For the working electrode, Prussian blue (PB) was first electrodeposited onto the Au electrode, followed by a drop-cast layer of selective membrane containing glucose oxidase (GO*x*) and carbon nanotubes, and finally topped with a drop-cast layer of Nafion (Fig. 2a, also see “Methods and materials” for details). In the presence of glucose, GO*x* catalyzes the following reaction:

$${\rm{glucose}}\,+\,{\rm{oxygen}}\,\mathop{\to }\limits^{{\rm{GO}}x}\,{\rm{hydrogen}}\,{\rm{peroxide}}\,+\,{\rm{gluconic}}\,{\rm{acid}}$$

The product species hydrogen peroxide (H_2_O_2_) is then reduced by the PB transducer, eliciting an amperometric response, which reflects the fluctuation in the glucose concentration (Fig. 2b). The amperometric response is recorded under a −0.1 V voltage posed by the A/D differential module relative to the reference electrode.

Two glucose sensor patches, one without (SP#1) and one with (SP#2) the topmost Nafion film, were first characterized in a semi-infinite diffusion environment (Fig. S3). The sensor patches were immersed in bulk solution (100 mL) and connected to an electrochemical workstation (Fig. S4). The CV curves and electrochemical responses remained stable in repeated experiments (Fig. S2). The amperometric responses of the glucose sensor patch (SP#2) reflect a linear diffusion pattern in bulk glucose solution with typical ISF glucose concentrations ranging from 0 to 200 μM (Fig. S5). The amperometric responses of SP#1 and SP#2 to glucose concentrations were measured at 1.40 and 2.42 nA/μM, respectively, indicating a 40% enhancement in sensor sensitivity in the presence of the Nafion film (Fig. 2c). The long-term stability study over a two-week period revealed a 15% decrease in sensitivity for SP#1 (1.40–1.06 nA/μM) and 8% for SP#2 (2.42–2.21 nA/μM) (Fig. 2c, d). Further analyses of the long-term stability study are shown in Fig. S6. The decay in the amperometric response of the Nafion-coated sensors was within 7.5% at all glucose concentrations tested. These results, together with the stronger absolute amperometric responses of SP#2, prove the advantages that Nafion modification delivers to the glucose sensors. The selectivity of SP#2 was further verified against other interfering components in ISF, such as lactic acid (LA) and hyaluronic acid (HA) (Fig. 2e). SP#2 also showed good reproducibility in repeated tests with standard glucose solutions (Fig. S7). The range of the 5 measured results of the same concentration was no larger than 7.6% of the mean value (for 20 µM) among all five concentrations tested.

**Fig. 3:**
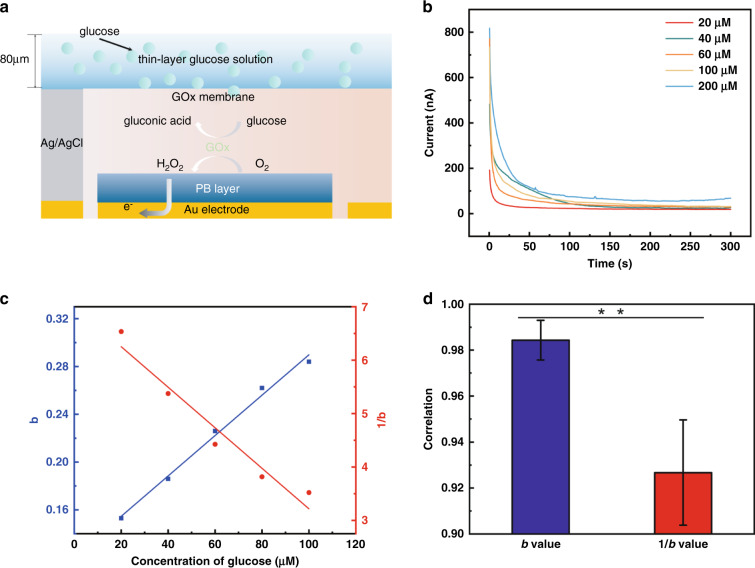
Performance test of glucose sensors in a small volume of solution. **a** Schematic diagram of glucose monitoring in the thin-layer electrochemical model. **b** Current–time behavior of the sensors with 4 μL of glucose solution at different concentrations. **c** Linear fit of *b* and 1/*b* against glucose concentration *C*. **d** Comparison of the correlation coefficients corresponding to the linear fits in (**c**). Data represent the mean ± s.d. of three replicates. ***p < 0.01 by Student’s t-test

In practical applications, the volume of biofluid between the biosensor and skin would be on the microscale (<5 µL). As a result, the capture of glucose by the GO*x* selective membrane is better described by a finite diffusion model, leading to a different chronoamperometric response pattern. Considering this deviation, SP#2 was further characterized in a microfluidic scenario. Four microliters of glucose solution was applied to the sensor electrodes, resulting in an initial thickness of approximately 80 μm (Fig. 3a). Then, the sensor patch was connected to the electrochemical workstation (Fig. S9). The chronoamperometric responses of the sensors decayed rapidly and reached a steady near-zero state within 200 s at all glucose concentrations from 0 to 200 μM (Fig. 3b), indicating the necessity of a calibration algorithm to correct the current-time behavior of the sensors with microvolume solutions. Herein, a calibration algorithm is proposed. Based on Fick’s second law^34^, the Cottrell equation under a semi-infinite boundary and the thin-layer electrochemical model^35,36^, the final output current *i(t)* can be expressed as:

$$i(t)=A\,{t}^{-b}$$

where *A* is a constant, and *b* is a value determined by the glucose concentration *C*. *b* is either directly proportional to *C* (*b = kC*) or inversely proportional to *C (b = k/C)*. The detailed derivation is given in the “Methods and materials” section. The linear fits of *b* and 1/*b* against different glucose concentrations were plotted with the experimental data (Fig. 3c), and the corresponding correlation coefficients (Fig. 3d) indicate that *b* has a better correlation when directly proportional to the glucose concentration (*C*) in ISF. The detailed data are given in Table S2. The final calibration algorithm is:

$$i(t)=A\,{t}^{-kC}.$$

### On-body testing

**Fig. 4:**
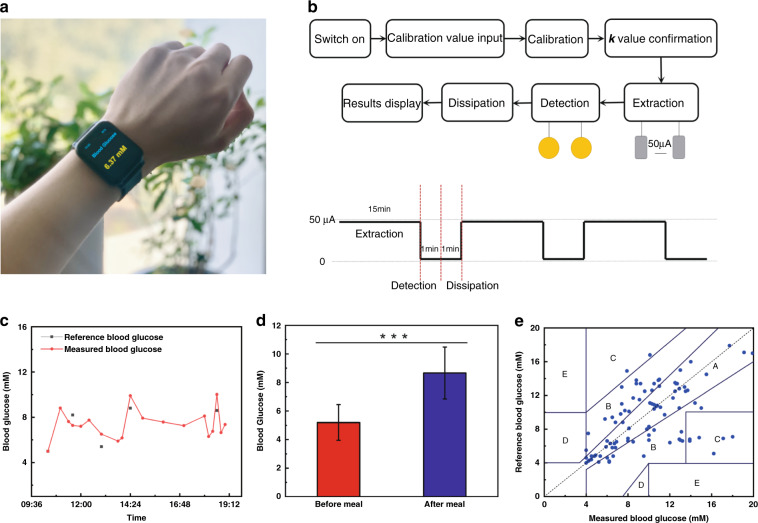
On-body test of the watch. **a** Photograph of a volunteer wearing the watch with blood glucose levels displayed in real time. **b** Workflow of the glucose-monitoring watch. **c** The blood glucose variation curve of a volunteer measured by the watch during the daytime compared to true blood glucose values (reference) obtained from finger blood. **d** Glucose concentrations before and after a meal measured by the watch from five volunteers. Data represent the mean ± s.d. of five replicates. ***p < 0.001 by Student’s t-test. **e** Plot of glucose concentrations measured from 23 volunteers by the watch and by a commercial glucose meter

For on-body testing, the glucose sensor patch was fixed on the inside of the watchband, and a volunteer was asked to wear the watch on the wrist (Fig. 4a and Fig. S10). The workflow of the watch system is illustrated in Fig. 4b. A calibration value obtained from a commercial glucose meter is first input into the system for the microcontroller to execute the calibration algorithm and confirm the constant value *k*. Then, an electric current is delivered to the extraction electrodes to run reverse iontophoresis for 15 min, and the microcontroller reads the signal output for 1 min to calculate the corresponding blood glucose level. To prevent interference from other extracted metabolites and electrolytes, the remaining ISF is allowed to dissipate for an additional 1 min. Finally, the numerical blood glucose result is displayed on the LED screen of the watch and sent to the user’s smartphone. One measurement cycle lasts for a total of 17 min, i.e., the user’s blood glucose level could be measured 4 times approximately every 1 h.

The volunteer wore the watch for approximately 10 h during the day, and the blood glucose fluctuation measured by the watch was recorded (Fig. 4c). To investigate the accuracy of glucose measurements by the watch, the volunteer’s blood glucose level was also measured four times by the fingerstick blood test using a commercial glucose meter (results indicated by black dots in Fig. 4c) for reference. All fingerstick blood tests except the second were performed immediately after meals. The blood glucose levels measured by the watch, especially the peaks, accurately reflected the trend of the volunteer’s true blood glucose levels. A two-volunteer (1 diabetic and 1 nondiabetic) trial was conducted to assess the accuracy of consecutive measurements by the watch. Five fasting glucose levels of each volunteer were measured by the watch within 1.5 h and compared to the accompanying finger-stick blood glucose test results. The two types of results matched well for both volunteers, indicating good accuracy and reproducibility of glucose measurements by the watch in the short term (Fig. S8). This result also serves as circumstantial evidence of the reproducibility of the iontophoresis function in the watch. We further tested the performance of the watch on five other volunteers, measuring their blood glucose levels before and after a meal. The watch successfully captured the increase in blood glucose levels after a meal (Fig. 4d). To evaluate the accuracy of glucose measurements by the watch with a widely acknowledged criterion, the Clarke error grid was plotted using the measurement results obtained from 23 volunteers (Fig. 4e), including 13 diabetes patients and 10 nonpatients. The results and statistics of measurement by the watch are presented in Fig. S11 and Table S3. The percentage of data points in zone A and zone B of the Clarke error grid, which represents clinically accepted accurate readings and acceptable moderate readings that would not lead to inappropriate treatments, indicates the accuracy of the tested glucose meter. Remarkably, no experimental data points fell in zone D or zone E, suggesting that the watch yields high-quality measurement results without misleading or false readings^37^. The data points are concentrated in zone A (46.99%) and zone B (37.35%), revealing an 84.34% overall accuracy of blood glucose tests conducted with the watch. Additionally, all volunteers reported a comfortable wearing experience resembling that of commercial smartwatches, with no obvious sensational difference (e.g., skin irritation) during glucose extractions. While the fitting of the watchband is not too tight, it is still able to secure the sensor patch to the user’s wrist. To verify that daily body motions do not impair the sensing performance of the watch, we compared the measurement results from two watches, one worn on a static arm and the other on a moving arm, of the same nondiabetic volunteer. The difference between the average results (of six measurements each) from the two watches was 2.1% (Fig. S12), comparable to the error of the same sensor between repeated measurements, indicating that daily body motions do not affect the performance of the watch.

## Conclusions

In summary, we developed a highly integrated glucose monitoring watch and achieved noninvasive continual blood glucose monitoring with clinically acceptable accuracy. Reverse iontophoresis-based ISF extraction by a flexible glucose sensor patch allows painless glucose detection, and the watch-like design ensures comfortable daily wear, facilitating continual glucose monitoring. Real-life testing of the watch on 23 volunteers revealed 84.34% clinical accuracy of blood glucose measurements, suggesting it is worth further improvement for commercialization. Subsequent efforts could be made in a few directions; for example, the accuracy could be improved by providing customized models to accommodate potentially interfering factors such as age, gender^38^, exercise^39^, and illness^40^. The PCB could be miniaturized and integrated into existing smartwatch models to create a truly noninvasive continuous glucose monitoring smartwatch.

## Methods and materials

### Materials

Glucose oxidase (GO*x*, from *Aspergillus niger*), chitosan, single-walled carbon nanotubes, iron(III) chloride, potassium ferricyanide(III), and 5% Nafion solution were purchased from Macklin. Acetic acid (2%), potassium chloride, and PBS (pH 7.2) were purchased from Aladdin, and 100 μm thick PI film was purchased from McMaster-Carr. All reagents were used as received.

### Fabrication of electrodes

The fabrication of the electrodes is illustrated in Fig. S1. First, the polyimide (PI) film was cleaned with acetone, ethanol, and ultrapure water. Then, the electrode and wire areas were defined by a photolithographed layer of positive photoresist (AZ1500). Subsequently, 50 nm Cr/80 nm Au was deposited via magnetron sputtering and lift-off in acetone. Finally, another layer of positive photoresist (AZ1500) was photolithographed onto the nonelectrode areas to insulate the wires.

### Preparation of glucose sensors

For the working electrodes, three modification steps were performed sequentially, coating the Au electrode with a Prussian blue (PB) layer, a GO*x* selective membrane, and a Nafion film. PB was electrodeposited onto the Au electrodes at 0.4 V (relative to an external reference electrode) for 30 s in a fresh solution containing 2.5 mM FeCl_3_, 100 mM KCl, 2.5 mM K_3_Fe(CN)_6_, and 100 mM HCl. For the selective membrane, chitosan was first dissolved in 2% acetic acid, and the resulting 1% chitosan solution was mixed with 2 mg/mL single-walled carbon nanotubes by ultrasonic agitation over 30 min to achieve viscous consistency. Then, 10 mg/mL GO*x* in PBS (pH 7.2) was mixed thoroughly into the chitosan/carbon nanotube mixture at a 1:2 v/v ratio. Fifteen microliters of the GO*x*-containing selective membrane material was drop-cast onto each PB/Au electrode, followed by 15 µL of 5% Nafion solution to drop-cast the Nafion film. For the reference and extraction electrodes, Ag/AgCl ink was screen-printed onto the Au electrodes. The designated counter electrodes were left unmodified. The sensor patches were stored at 4 °C in the dark, and the solutions were also stored at 4 °C when not in use.

### Calibration algorithm

For ISF glucose detection, where the fluid between the sensors and the skin surface is continuously consumed, the thickness of the fluid decreases dynamically and is therefore expressed as a function of time, $l(t)$. Throughout the measurement process, the diffusion of glucose follows Fick’s second law^34^:

1 $$\frac{\partial {C}_{g}(x,t)}{\partial t}={D}_{m}\left[\frac{{\partial }^{2}{C}_{g}(x,t)}{\partial {x}^{2}}\right]$$

where *D*_*m*_ is the mass diffusive coefficient and *C*_*g*_ is the glucose concentration.

As glucose is rapidly consumed in the extracted ISF, the mass transfer pattern quickly switches from a semi-infinite diffusion model to a finite diffusion model, i.e., $l(t)\,$decreases and ${({D}_{m}t)}^{1/2}$ increases with detection time *t*.

Taking semi-infinite diffusion and the boundary effect into account^36^, the following equation is obtained using the Laplace transform:

2 $${\kappa }_{1}{C}_{g}(x,t)={\kappa }_{1}{C}_{g}^{\ast }{\rm{erf}}\frac{x}{2{D}_{m}^{1/2}{t}^{1/2}}$$

where ${C}_{\!g}^{\ast }$ is the initial glucose concentration at *t* = 0.

In addition, considering the decline rate of $l(t)\,$ and detection time *t*, the approximate $i(t)\,$ can be expressed as:

3 $$\begin{array}{ll}i(t)={\kappa }_{1}\frac{nF{A}_{m}{D}_{m}^{1/2}{C}_{g}^{\ast }}{{\pi }^{1/2}}{t}^{-1/2}\\ \qquad\quad+\,{\kappa }_{2}\frac{4nF{A}_{m}{C}_{g}^{\ast }{D}_{m}}{\pi l}{\rm{exp}}\left(-\frac{{\pi }^{2}{D}_{m}}{{l}^{2}}t\right)\end{array}$$

${\kappa }_{1},{\kappa }_{2}$ are variables dependent on $l(0)\,$and the concentration distribution of electroactive species. The switching of one of them from 1 to 0 and the other from 0 to 1 represents the complete switching of the diffusion model applied, i.e., ${\kappa }_{1}=1,{\kappa }_{2}=0$ depicts the current responses in a semi-infinite diffusion model^35^, and ${\kappa }_{1}=0,{\kappa }_{2}=1$ depicts the current responses in a thin layer electrochemical model. Based on the equations above, $i(t)\,$ can be further simplified. Combined with theoretical analysis and experimental data, $i(t)$ can be expressed as:

4 $$i(t)=A\,{t}^{-b}$$

### Circuit design

The PCB circuit is based around the STM32L412K8 12-bit microcontroller (Texas Instruments) (module 3 in Fig. 1b), which connects a constant current source, an A/D differential module, a Bluetooth module, and a power supply (modules 1, 2, 4 and 5 in Fig. 1b). In the schematic diagram of the microcontroller interface, PA1 and PA5 are connected to the working electrodes for amperometric signal reading, and PA8 is connected to the constant current source for current delivery for reverse iontophoresis (Fig. S13). The Bluetooth chip is connected to pins PA2 and PA3 of the microcontroller to achieve wireless transmission to a cell phone. Within the signal conditioning circuit, a +1.25 V external voltage is applied as the noninverting input of the operational amplifier, and the amperometric signals from the working electrodes serve as the inverting input. The signals are further transmitted and processed by the filter circuitry (Fig. S14a). On the sensor interface, pins 1, 5, 6, and 12 correspond to the extraction electrodes; pins 2 and 11 correspond to the counter electrodes; pins 3 and 10 correspond to the working electrodes; and pins 4 and 7 correspond to the reference electrodes (Fig. S14b).

### Mobile application design

A mobile application was designed for a better user experience. As shown in Figs. 1e and S10, the interface of the application consists of several modules, enabling users to check the connection status between the watch and the user’s smartphone, input the calibration value, and read the real-time blood glucose measurement result. In addition, the application is capable of storing historic data and plotting the trend of blood glucose over the period of wearing.

### Volunteer on-body experiment

The on-body testing of the watch was performed in compliance with the protocol that was approved by the institutional review board of China-Japan Friendship Hospital (2021-112-K70). Thirteen diabetic patients aged 40–60 were recruited from China-Japan Friendship Hospital, and 10 nonpatients aged 20–40 were recruited within Beihang University. The subjects’ wrists were cleaned and dampened with PBS (pH 7.2) to maintain the pH. Each subject wore the watch for 8 h (from 10:00 to 18:00). Six fingerstick blood samples were taken from each subject and measured by a commercial glucose meter (Accusure 580, Yuwell Co., Ltd.) to serve as a reference in the Clarke error grid accuracy assessment; one was used for calibration, two were taken 1 h after a meal (lunch and dinner, each), and the other three were taken at random time points. The values obtained with the commercial glucose meter and with our watch were recorded and further analyzed.

To test the reproducibility of the reverse iontophoresis function, we carried out volunteer trials. Two volunteers (1 diabetic patient and 1 nonpatient) were asked to wear the watch in a static position between 15:00 and 16:30 in the afternoon. Neither volunteer ingested food within the 3 h preceding the trial so that their blood glucose remained relatively stable. Each watch was able to run 5 blood glucose tests during the 1.5 h period, and an accompanying fingerstick test (Accusure 580, Yuwell Co., Ltd.) was performed for each volunteer at each time point when the watch ran its glucose measurement.

We conducted further experiments to verify that body motion did not cause inaccurate test results. A nondiabetic volunteer wore a glucose detecting watch on each wrist. During the test, the subject’s left arm remained still, and the right arm made constant movements (e.g., arm swings, arm flexions, wrist movements). During the 2 h experiment, six measurements by each watch and 6 accompanying finger-stick blood glucose test results were recorded.

## Supplementary information

Highly Integrated Watch for Non-invasive Continual Glucose Monitoring_supporting (revised)

Highly Integrated Watch for Non-invasive Continual Glucose Monitoring_supporting (revised marked)
