# Supplementary material for: Highly integrated watch for noninvasive continual glucose monitoring
Source: Microsyst Nanoeng. 2022 Feb 23;8:25. doi: 10.1038/s41378-022-00355-5 (PMC8866463; doi:10.1038/s41378-022-00355-5)
Supplement: Supplementary file 1 — Highly Integrated Watch for Non-invasive Continual Glucose Monitoring_supporting (revised) [file 41378_2022_355_MOESM1_ESM.docx]

**Highly Integrated Watch for Non-invasive Continual Glucose Monitoring**

Tianrui Chang ^a,1^, Hu Li ^a,b,1^, Nianrong Zhang ^c^, Xinran Jiang ^a^, Xinge Yu ^b^, Qingde Yang ^d^, Zhiyuan Jin ^a^, Hua Meng ^c*^ and Lingqian Chang ^a*^

^a^ Beijing Advanced Innovation Center for Biomedical Engineering, Key Laboratory for Biomechanics and Mechanobiology, School of Biological Science and Medical Engineering, Beihang University, Beijing 100083, China;

^b^ Department of Biomedical Engineering, City University of Hong Kong, Hong Kong, China

^c^ General Surgery Department & Obesity and Metabolic Disease Center, China-Japan Friendship Hospital, Beijing, China, 100029, China

^d^ Sense Future (HangZhou) Co., Ltd, Hangzhou, 311217, China

^1^ These authors contribute equally to this work

* Corresponding Emails: [lingqianchang@buaa.edu.cn](mailto:lingqianchang@buaa.edu.cn); menghuade@hotmail.com

***Contents***

**Supplementary Figures**

**Figure S1**. Fabrication process of the electrodes of the glucose sensor patch.

**Figure S2**. Electrochemical characterization of the glucose sensor patches.

**Figure S3**. Glucose sensor patches without or with Nafion modification.

**Figure S4**. Test of glucose sensors in bulk solution.

**Figure S5**. Correlation of amperometric responses of glucose sensor patch SP#2 with t^-1/2^ in glucose solution of different concentrations.

**Figure S6**. Long-term stability of the glucose sensor.

**Figure S7**. Reproducibility of glucose measurement by one SP#2.

**Figure S8**. Match of glucose measurements by the watch to finger-stick blood glucose tests.

**Figure S9**. Test of glucose sensors under micro-volume solution.

**Figure S10**. Real-time blood glucose monitoring.

**Figure S11.** Results of glucose monitoring from 23 volunteers.

**Figure S12.** Effect of body motion on sensor performance of the watch.

**Figure S13.** Schematic diagram of the microcontroller interface.

**Figure S14.** Circuit design.

**Supplementary Tables**

**Table S1**. Comparison between our device and previously published iontophoresis-based glucose meters

**Table S2**. Values of *b* and *1/b* under different glucose concentrations.

**Table S3**. Blood glucose differences between diabetic patients and non-patients.

***Supplementary Figures***


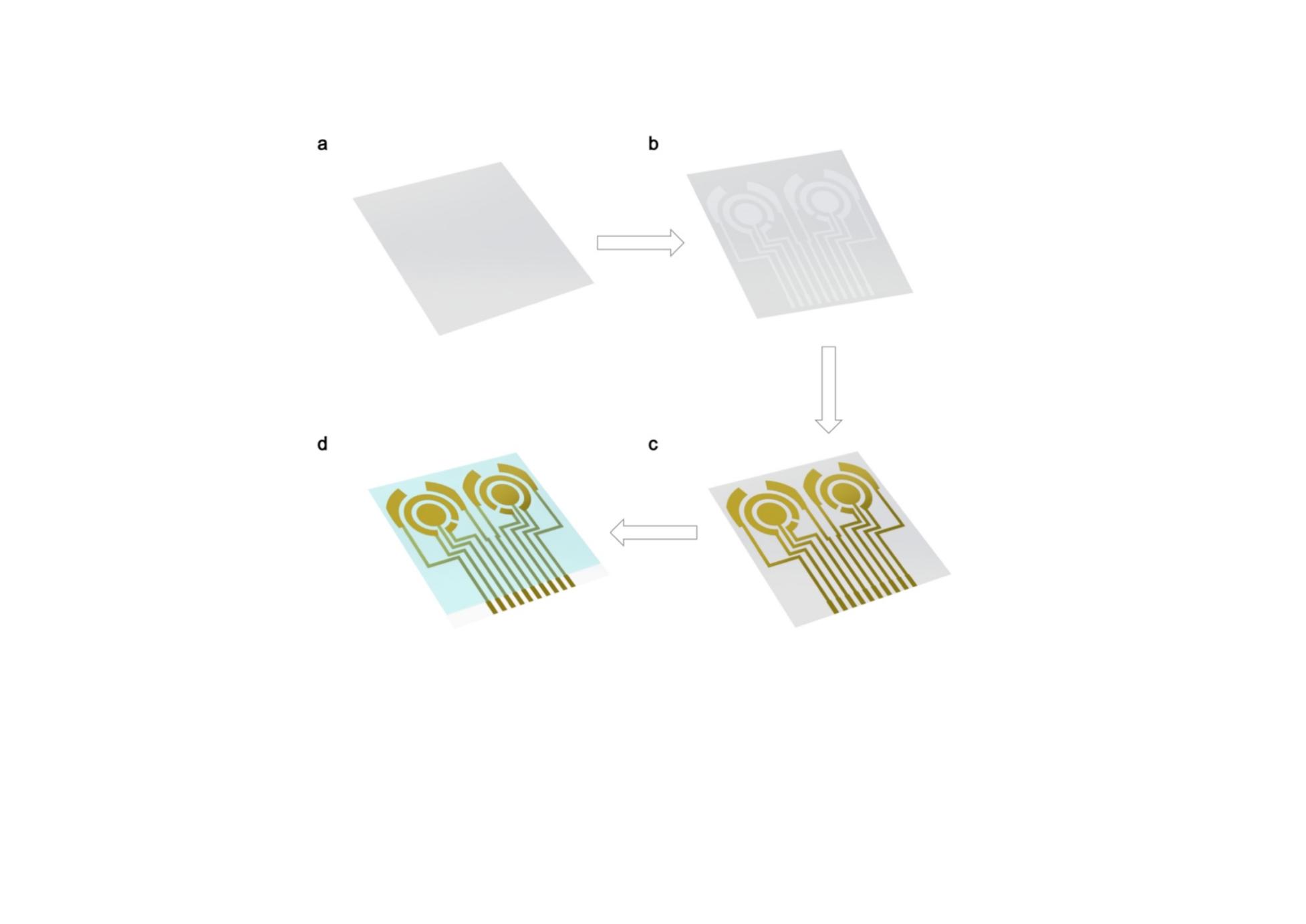


**Figure S1.** **Fabrication process of the electrodes of the glucose sensor patch.** (a) Cleaned polyimide (PI) film. (b) Electrode area patterned by photolithography using positive photoresist. (c) Au sputter deposited in the preserved area. (d) Non-electrode area insulated with another layer of photolithographed positive photoresist.


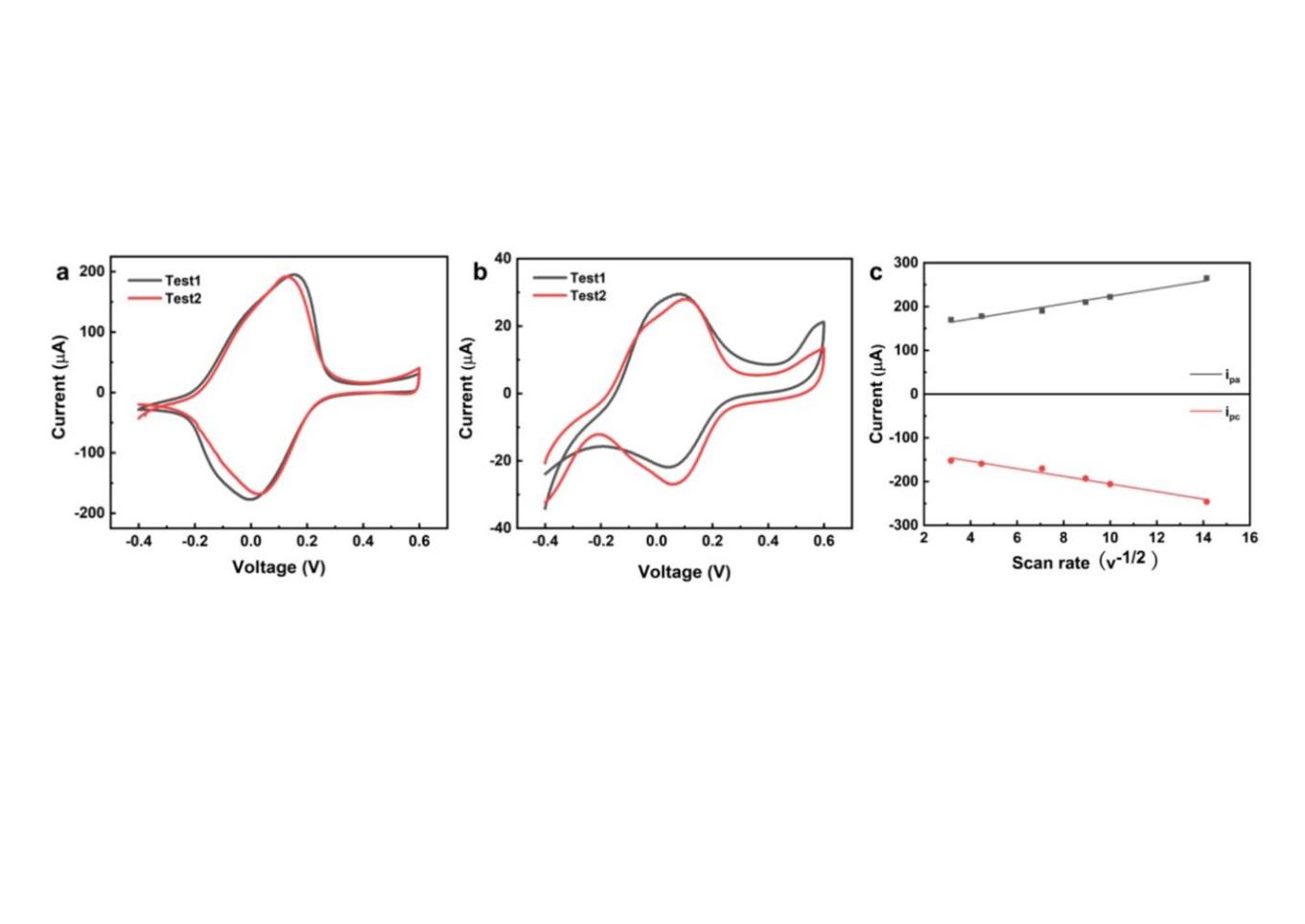


**Figure S2. Electrochemical characterization of the glucose sensor patches.** (a) CV curves of two sensor patches in PBS before GOx modification. (b) CV curves of the same two sensor patches in PBS after GOx modification (without Nafion modification). (c) Linear fit of *i_pa_* and *i_pc_* under different scan rates.


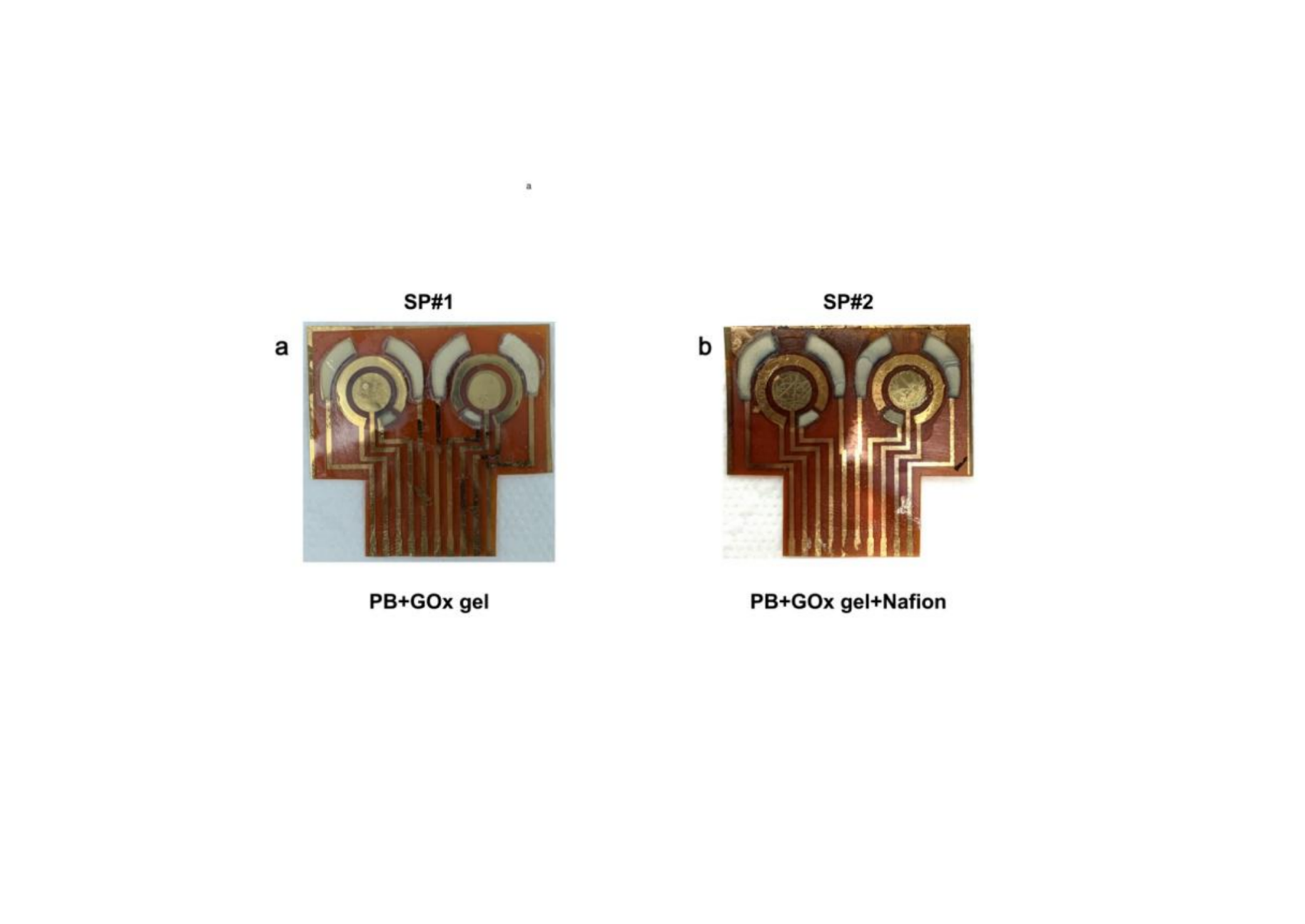


**Figure S3.** **Glucose sensor patches without or with Nafion modification.** (a) The working electrodes of sensor patch #1 (SP#1) were modified with Prussian blue (PB) mediator and glucose oxidase (GOx)-containing selective membrane. (b) The working electrodes of SP#2 were further coated with a Nafion film in addition to the PB and GOx layers. The Nafion film enhances sensor sensitivity as well as preserving it over long periods of time by preventing GOx loss.


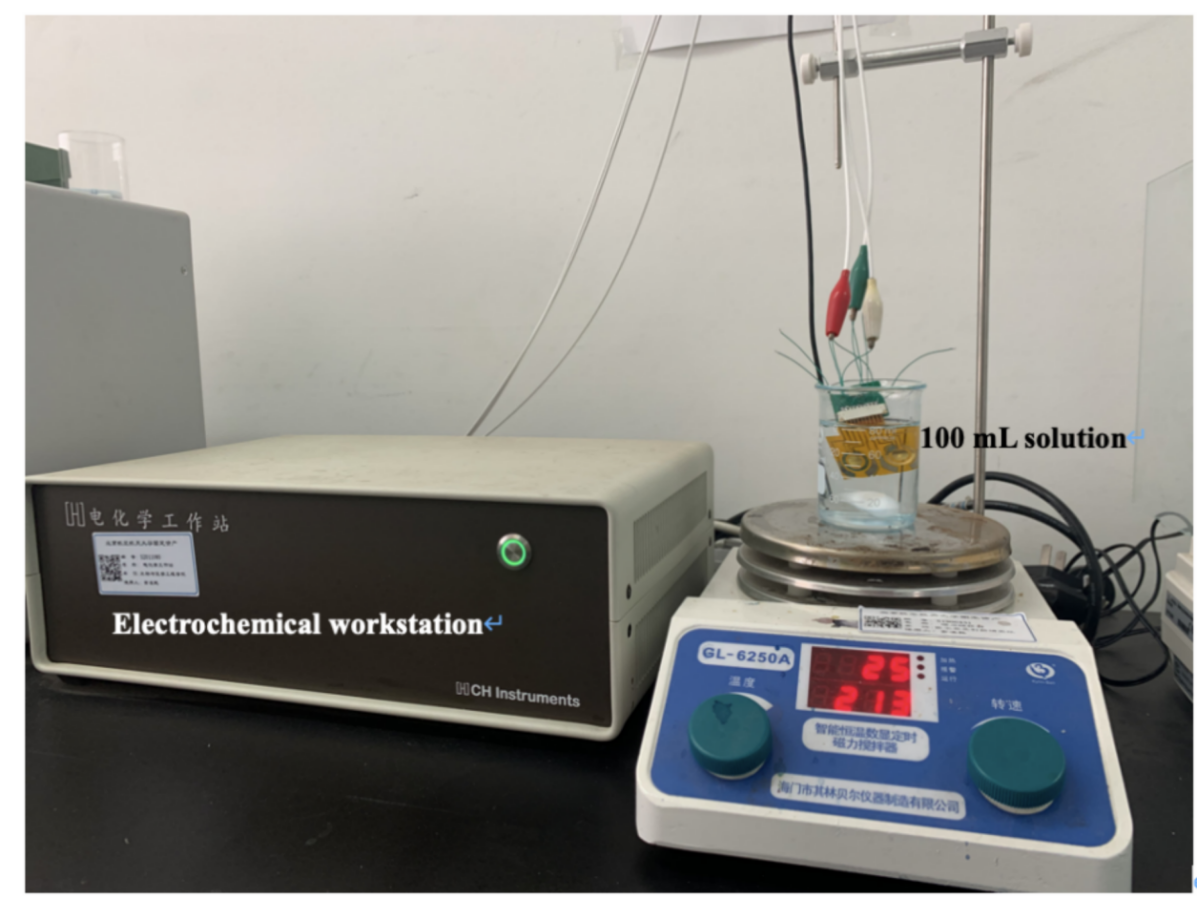


**Figure S4. Test of glucose sensors in bulk solution.** A glucose sensor patch is first immersed in 100 mL PBS solution and connected to an electrochemical workstation. Glucose standard solution is then continuously added in and magnetically stirred.


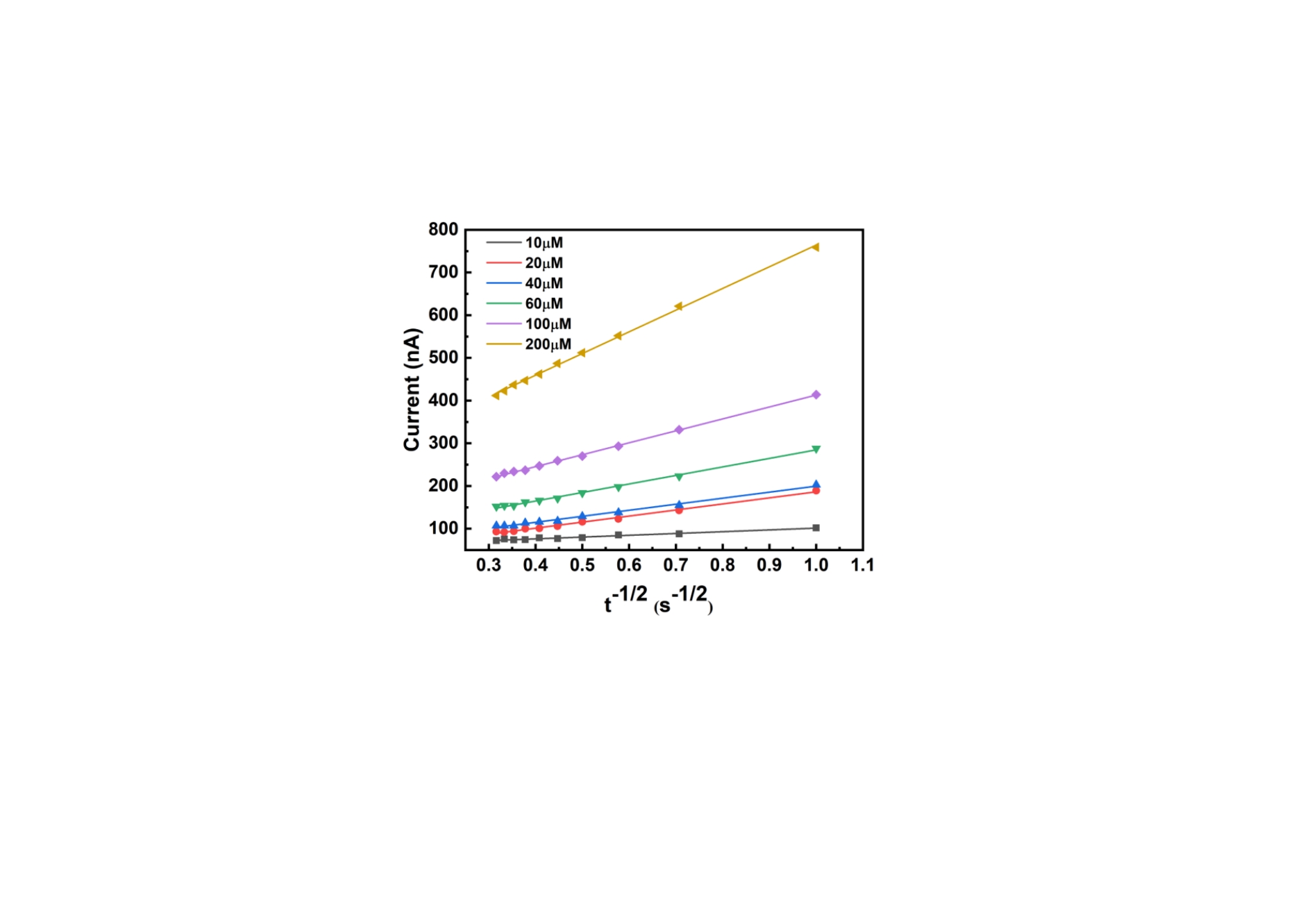


**Figure S5. Correlation of amperometric responses of glucose sensor patch SP#2 with t^-1/2^ in glucose solution of different concentrations.** The goodness of fit (R^2^) is above 0.99 at all physiologically relevant concentrations (10 µM to 200 µM), indicating that the sensor responses conform to the Cottrell equation under semi-infinite diffusion.


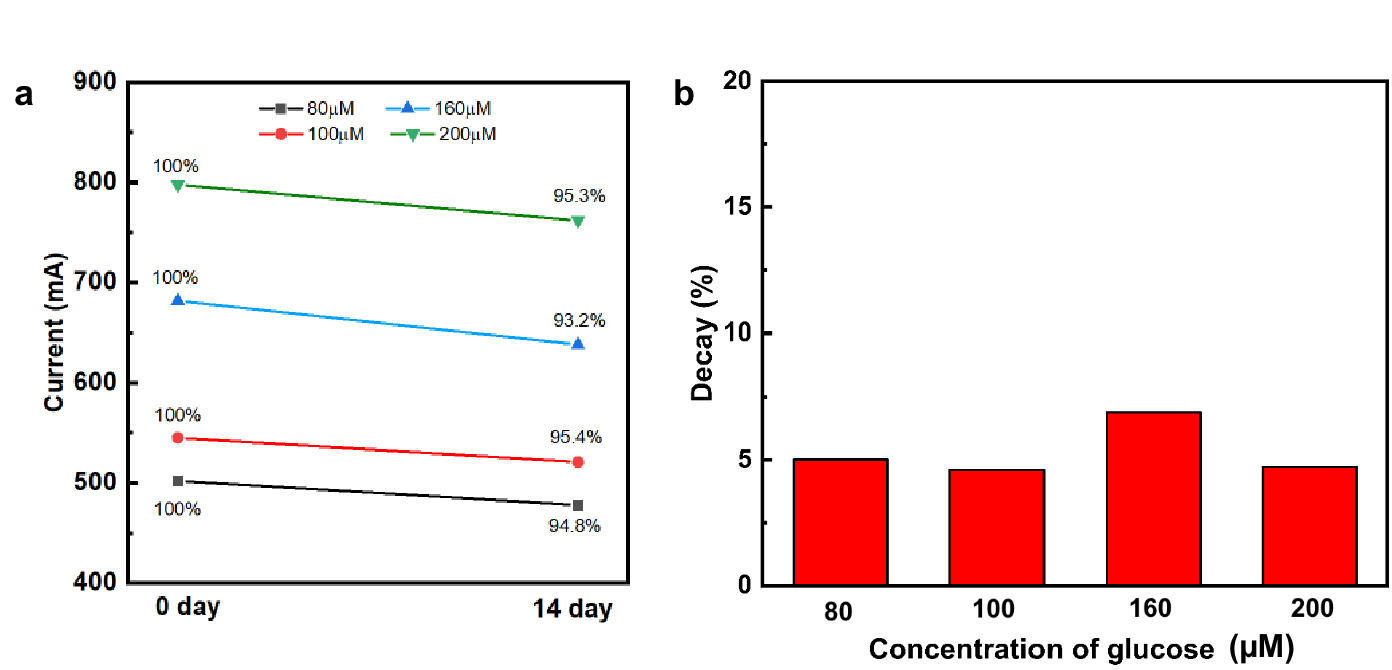


**Figure S6. Long-term stability of the glucose sensor.** (a) Average amperometric response of 3 newly fabricated sensors and that of the same set of sensors after two-week storage at 4℃. The sensors were tested under glucose concentration conditions from 80 µM to 200 µM. (b) Percentage current decay of the glucose sensors under glucose concentration of 80 µM to 200 µM after two-week storage. The current decay is 5.2% at 80 µM, 4.6% at 100 µM, 6.8% at 160 µM, and 4.7% at 200 µM, which were all within 7.5%, indicating good long-term stability of the glucose sensors.


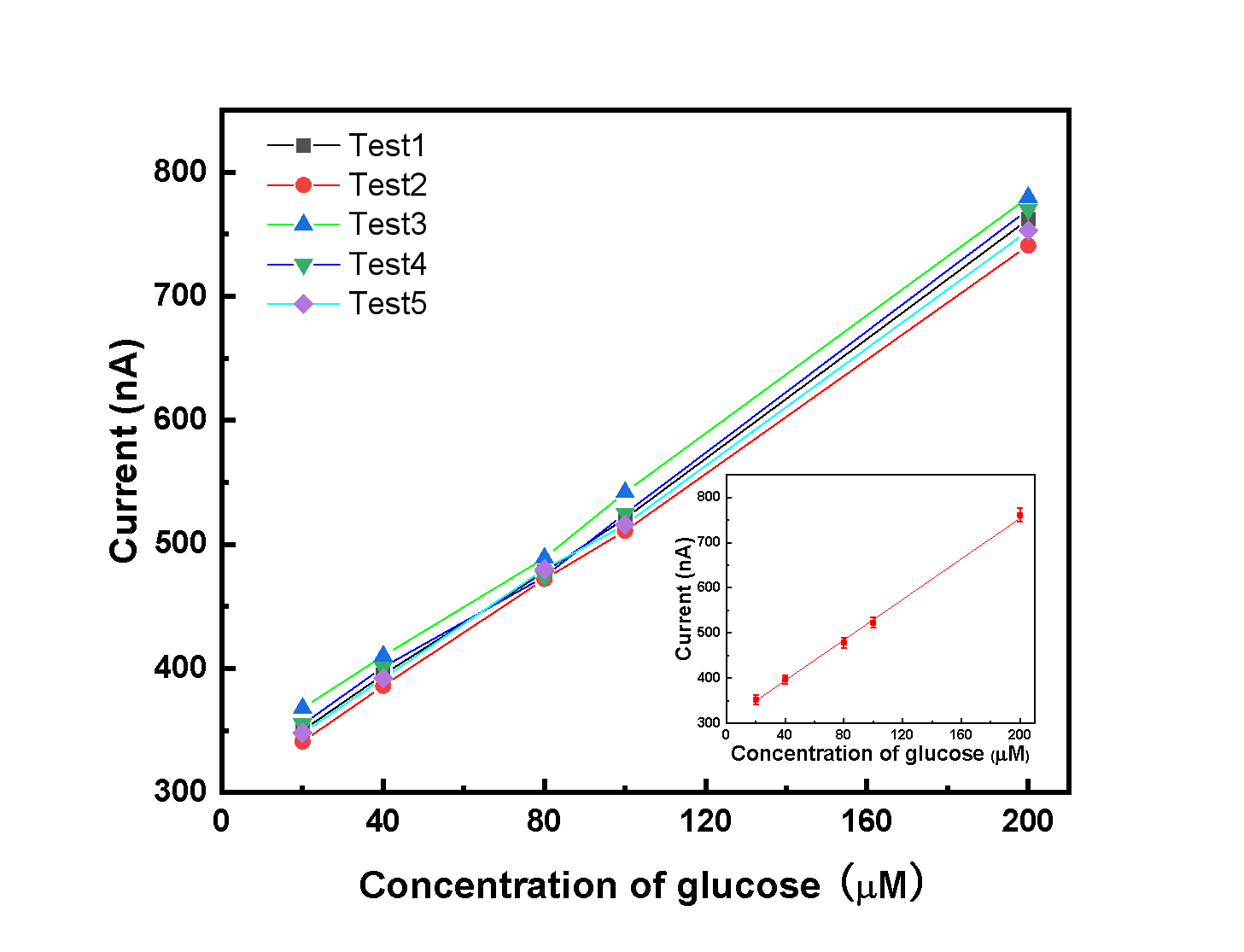


**Figure S7. Reproducibility of glucose measurement by one SP#2.** The amperometric response of the biosensor was tested five times in each concentration of standard glucose solution (20 µM, 40 µM, 80 µM, 100 µM and 200 µM). Inset plots the mean of the five tests with error bar at each concentration and the linear regression; data represent mean ± s.d. of 5 replicates.

***
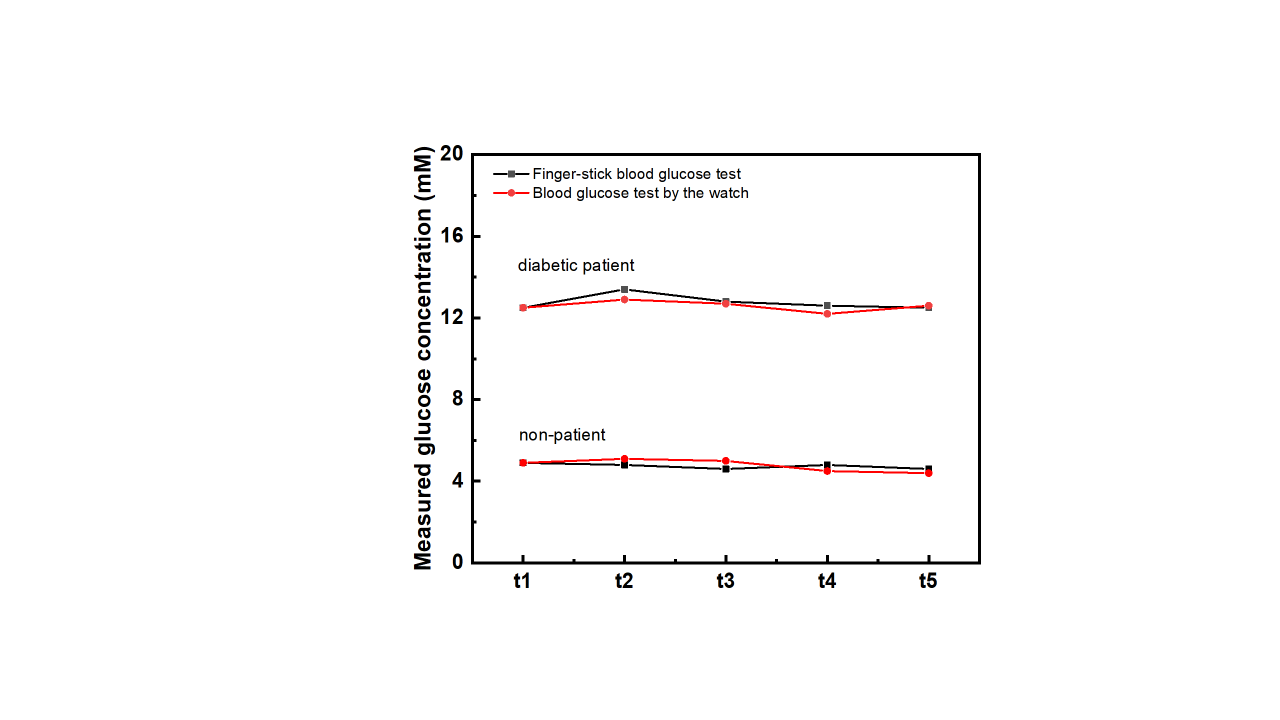
***

**Figure S8. Match of glucose measurements by the watch to finger-stick blood glucose tests.** 2 volunteers (1 diabetic patient and 1 non-patient) wore the watch in static position for 1.5 h. Neither volunteer had food ingestion within the 3 h preceding the experiment so that their blood glucose remained relatively stable. At each time point of glucose measurement by the watch (17 min apart), a finger-stick blood glucose test was also performed. The close match between the two type of results demonstrates the reproducibility of the iontophoresis function in the watch circumstantially.

**
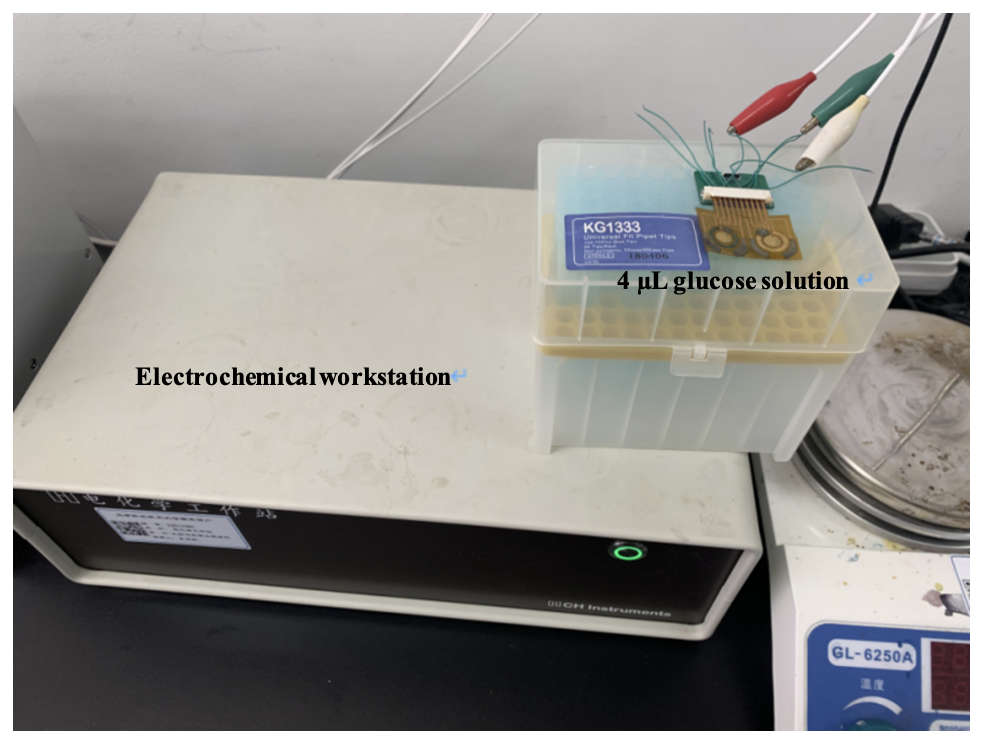
**

**Figure S9. Test of glucose sensors under micro-volume solution.** 4 µL glucose solution is dropped onto the sensors to simulate the fluid between the sensors and skin surface in actual use, and the sensor patch is connected to the electrochemical workstation.

***
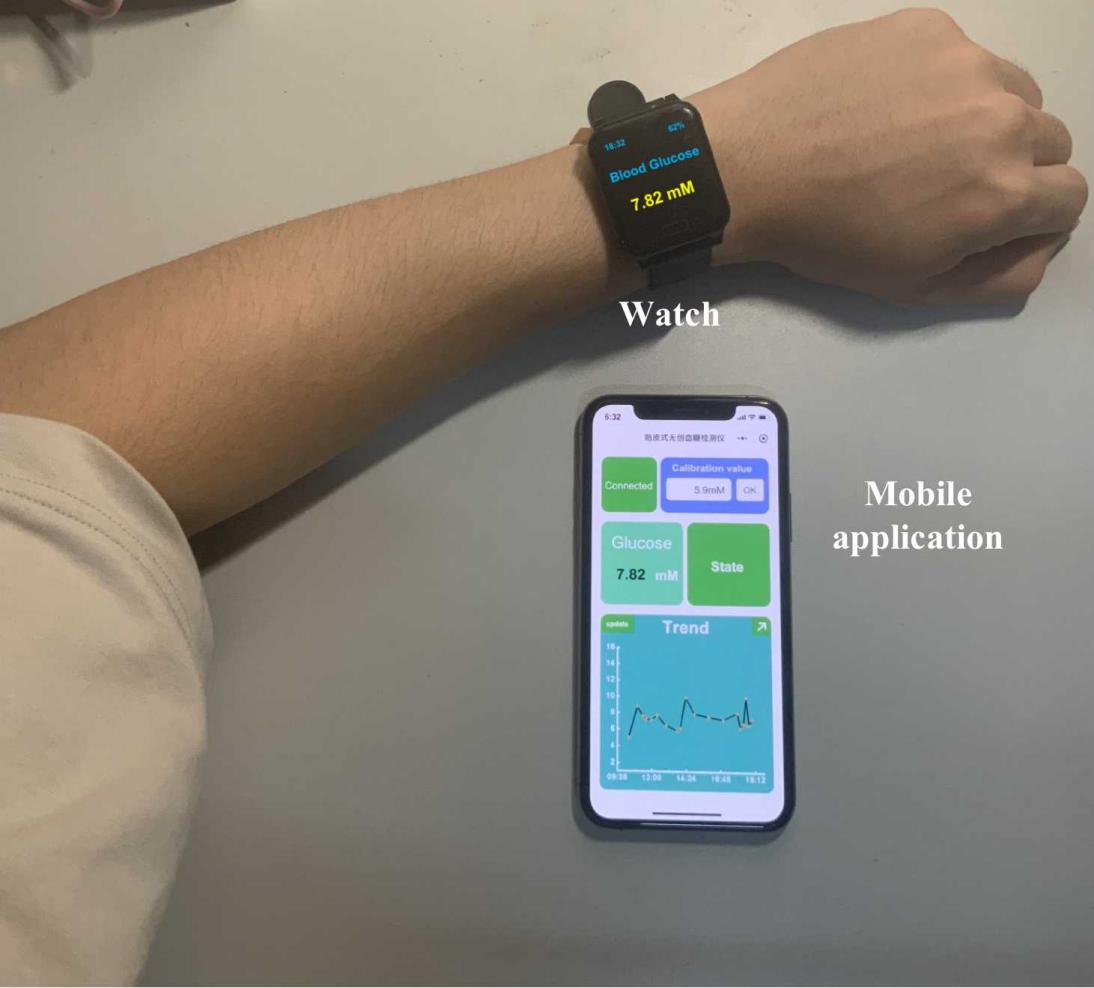
***

**Figure S10. Real-time blood glucose monitoring.** The newly updated blood glucose level is shown on the watch as well as the mobile application on user’s smartphone. The mobile application is designed to show the connection status between the watch and user’s smartphone (“Connected”), a box for calibration value input, the real-time measurement result of blood glucose level, a clickable box where user could check the status of the watch (i.e., which phase of the measurement cycle the watch is in), and user’s blood glucose fluctuation over the period of wearing. In this case, the volunteer has been wearing the watch for 10 hours (approximately from 9:30 to 19:30), and the fluctuation curve accurately reflects the three postprandial peaks of blood glucose level.

***
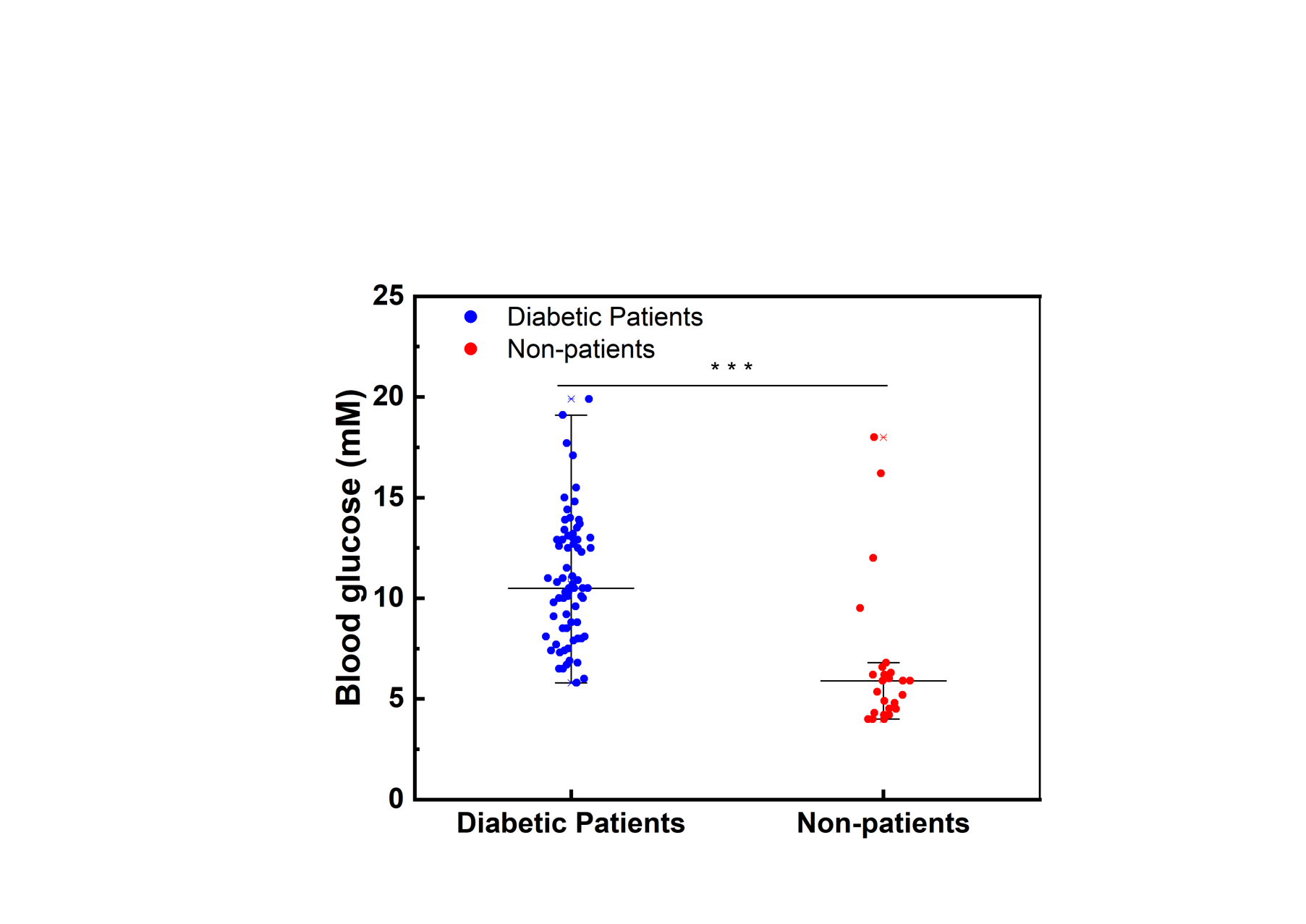
***

**Figure S11. Results of glucose measurement from 23 volunteers.** Of the 23 volunteers, 13 were diabetic patients and 10 were non-patients. The scatterplot shows the results of each test, and the results of the two groups (diabetic patients and non-patients) were significantly different. *** p < 0.001 by Student’s t-test.


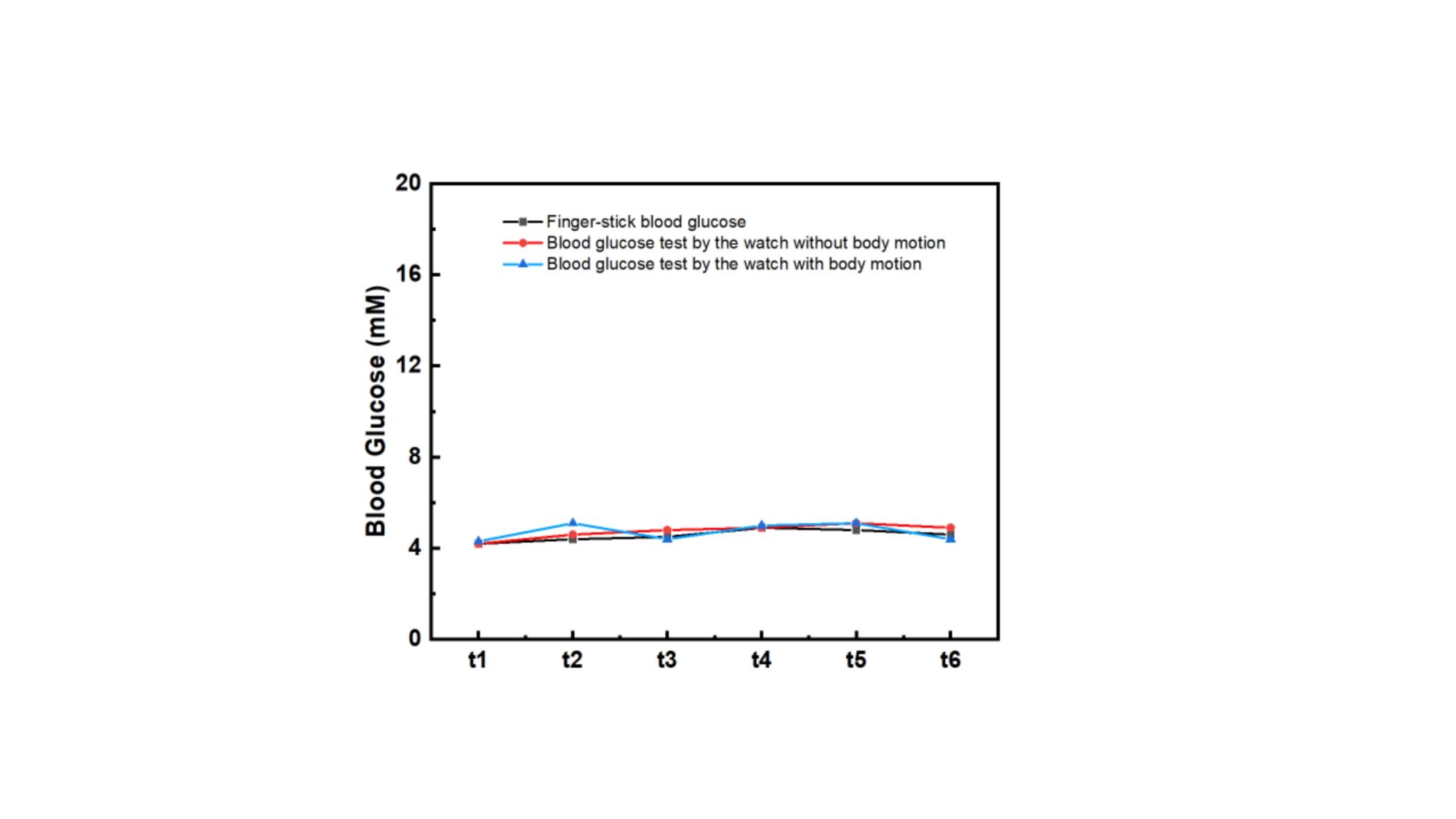


**Figure S12. Effect of body motion on sensor performance of the watch.** A non-diabetic volunteer wore one glucose detecting watch on their left and right wrist, respectively. During the test, the left arm remained still and the right arm kept making movements (e.g., arm swings, arm flexions, wrist movements). The average glucose concentration of 6 consecutive measurements by the two watches were 4.8 mM and 4.7 mM respectively for the still arm and the moving arm, which were 5.2% and 3.1% different from the average finger-stick blood glucose test results taken during the same period. The difference between the average results from the two watches stay within 2.1%, which is comparable to the error of a same sensor between repeated measurements, indicating that body motions do not show an impact on the sensing performance.


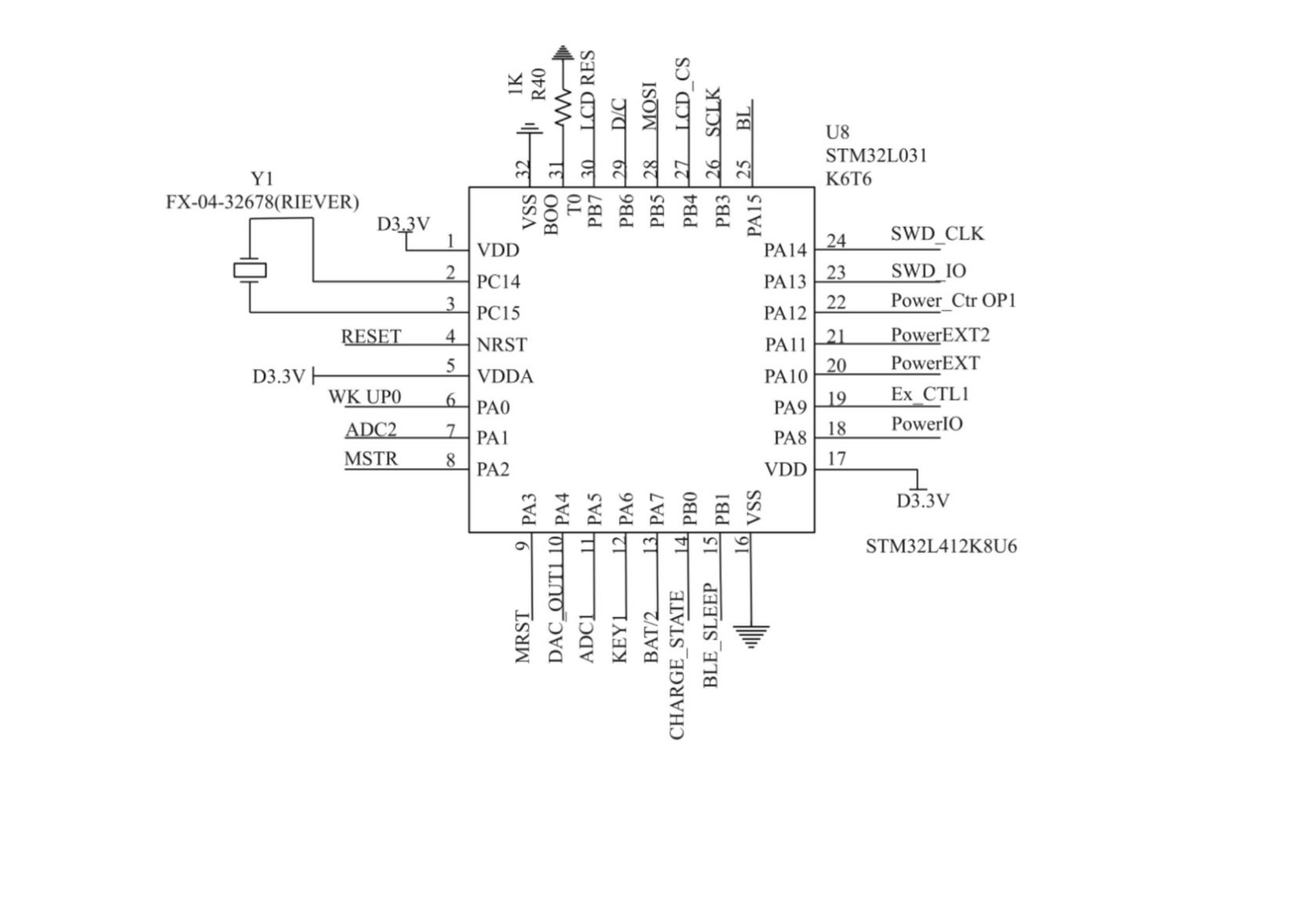


**Figure S13. Schematic diagram of the microcontroller interface.**


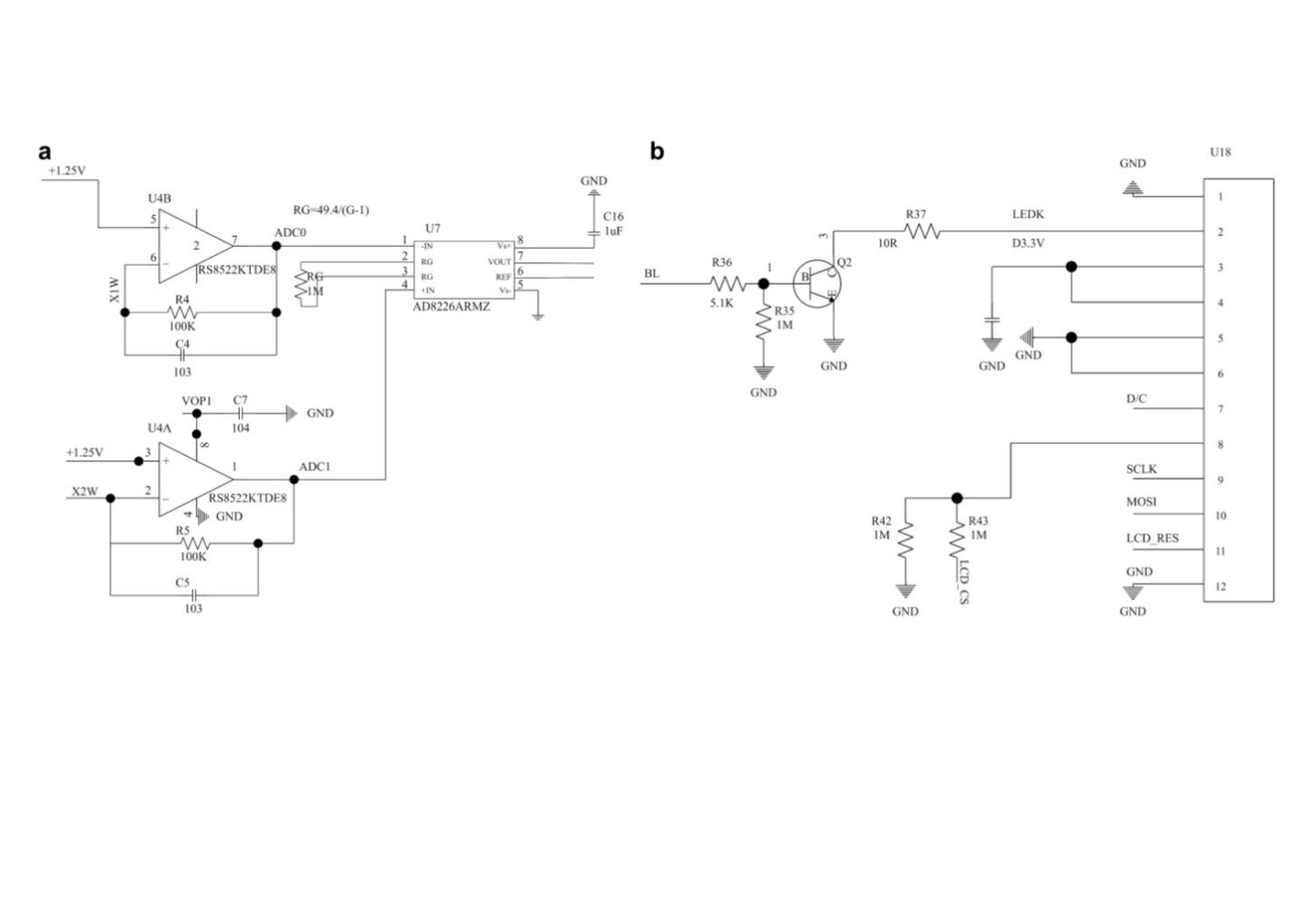


**Figure S14. Circuit design.** (a) Schematic diagram of the signal conditioning circuit. (b) Schematic diagram of the sensor interface.

***Supplementary Tables***

**Table S1**. Comparison between our device and previously published iontophoresis-based glucose meters

| Sample | Sampling  Method | Integration  Design | Sensitivity | Qualitative/  Quantitative | Ref. |
| --- | --- | --- | --- | --- | --- |
| ISF | Reverse iontophoresis | Watch | 2.42 nA µM^-1^ | Quantitative | This work |
| Sweat | Iontophoresis/ Exercise | Watch | 3.29 nA µM^-1^ | Qualitative | Zhao et al. (2019) ^1^ |
| Sweat | Iontophoresis | None | ~13.3 nA µM^-1^ | Quantitative | Kim et al. (2018) ^2^ |
| ISF | Reverse iontophoresis | Skin patch | 158.0 µA mM^-1^ | Quantitative | Chen et al. (2017) ^3^ |
| ISF | Reverse iontophoresis | None | ~48 nA mM^-1^ | Quantitative | Pu et al. (2021) ^4^ |
| ISF | Reverse iontophoresis | None | ~2.2 µA mM^-1^ cm^-2^ | Quantitative | Lipani et al. (2018) ^5^ |
| ISF | Reverse iontophoresis | Watch | - | Quantitative | Tierney et al. (2001) ^6^ |

**Table S2.** Values of *b* and 1*/b* under different glucose concentrations

| Glucose Concentration (µM) | b | 1/b |
| --- | --- | --- |
| 20 | 0.153 | 6.536 |
| 40 | 0.186 | 5.376 |
| 60 | 0.226 | 4.425 |
| 80 | 0.262 | 3.817 |
| 100 | 0.284 | 3.521 |
| 200 | 0.311 | 3.215 |

**Table S3.** Blood glucose differences between diabetic patients and non-patients.

| Subgroups | Average Blood Glucose (mM) | Fasting Blood Glucose (mM) | Postprandial Blood Glucose (mM) |
| --- | --- | --- | --- |
| Diabetic Patients | 10.96 | 8.99 | 14.42 |
| Non-patients | 6.62 | 5.01 | 8.33 |

**Reference**

1. Zhao, J. , Lin, Y. , Wu, J. , Nyein, H. , & Javey, A. A fully integrated and self-powered smartwatch for continuous sweat glucose monitoring. *ACS Sensors*, **4,** 1925–1933 (2019).

2. Jayoung, K. , Sempionatto, J. R. , Somayeh, I. , Hartel, M. C. , Abbas, B. , & Guangda, T. , et al. Simultaneous monitoring of sweat and interstitial fluid using a single wearable biosensor platform. *Advanced Science*, **5**, 1800880 (2018).

3. Chen, Y. , Lu, S. , Zhang, S. , Li, Y. , Qu, Z. , & Chen, Y. , et al.. Skin-like biosensor system via electrochemical channels for noninvasive blood glucose monitoring. *Science Advances*, **3**, e1701629 (2017).

4. Pu, Z. , Zhang, X. , Yu, H. , Tu, J. , & Li, D. A thermal activated and differential self-calibrated flexible epidermal biomicrofluidic device for wearable accurate blood glucose monitoring. *Science Advances*, **7**, eabd0199 (2021).

5. Lipani, L. , Doungmene, F. , Guy, R. H. , & Marken, F. Non-invasive, transdermal, path-selective and specific glucose monitoring via a graphene-based platform. *Nature Nanotechnology*, **13,** 504-511 (2018).

6. Tierney, M. J. , Tamada, J. A. , Potts, R. O. , Jovanovic, L. , & Garg, S. Clinical evaluation of the glucowatch biographer: a continual, non-invasive glucose monitor for patients with diabetes. *Biosensors & Bioelectronics*, **16**, 621-629 (2001).
